# Trends and Disparities in the Burden of Chronic Kidney Disease due to Type 2 Diabetes in China From 1990 to 2021: A Population‐Based Study

**DOI:** 10.1111/1753-0407.70084

**Published:** 2025-04-23

**Authors:** Yifei Wang, Shiya Gu, Zhixuan Xie, Zhiyong Xu, Wenfang He, Yexiang Chen, Juan Jin, Qiang He

**Affiliations:** ^1^ Department of Nephrology The First Affiliated Hospital of Zhejiang Chinese Medical University (Zhejiang Provincial Hospital of Chinese Medicine) Hangzhou Zhejiang China; ^2^ Zhejiang Key Laboratory of Research and Translation for Kidney Deficiency‐Stasis‐Turbidity Disease Hangzhou Zhejiang China; ^3^ Institute of Chronic Nephropathy Wenzhou Medical University Wenzhou Zhejiang China; ^4^ Department of Nephrology The First Affiliated Hospital of Wenzhou Medical University Wenzhou Zhejiang China; ^5^ Department of Nephrology, XianJu People's Hospital, Zhejiang Southeast Campus of Zhejiang Provincial People's Hospital Affiliated Xianju's Hospital, Hangzhou Medical College Xianju Zhejiang China; ^6^ The Third Clinical Medical College Zhejiang Chinese Medical University Hangzhou Zhejiang China

**Keywords:** China, chronic kidney disease due to type 2 diabetes mellitus, disease burden, risk factors, trends

## Abstract

**Background:**

This study analyzes the trends in the burden of chronic kidney disease due to type 2 diabetes (CKD‐T2D) in China from 1990 to 2021, evaluates variations in risk factors, and projects the disease burden through 2036.

**Method:**

Estimates of prevalence, incidence, mortality, and disability‐adjusted life years (DALYs) for CKD‐T2D were retrieved along with their 95% uncertainty intervals (UIs). Age‐period‐cohort analysis was used to assess burden trends from 1990 to 2021, identify risk factor population attributable fractions (PAFs), and project the burden through 2036.

**Results:**

In 2021, there were 20 911 520 CKD‐T2D cases in China, with an age‐standardized prevalence rate (ASPR) of 1053.92 per 100 000, an incidence rate (ASIR) of 23.07, an age‐standardized mortality rate (ASMR) of 5.72, and an age‐standardized DALY rate (ASDR) of 122.15. Although the overall burden showed a slow decline from 1990 to 2021, incidence continued to rise. The 2021 data revealed a marked age effect, with the burden rising with age. Period effects also contributed to an increased risk, with metabolic risk factors such as high fasting plasma glucose and BMI contributing the most. Projections suggest a decline in mortality and DALYs by 2036, while incidence will keep increasing.

**Conclusion:**

Despite declines in ASMR and ASDR, CKD‐T2D incidence and cases continue to rise, especially among males and the elderly. This increasing burden is driven by aging and metabolic risk factors. Early screening, education, and risk management are essential for addressing CKD‐T2D in China.

## Graphical abstract

This study analyzed the burden of chronic kidney disease due to type 2 diabetes (CKD‐T2D) in China from 1990 to 2021 and projected its trends through 2036. The overall disease burden of CKD‐T2D exhibited a gradual decline during 1990–2021, while the incidence continued to rise. Notably, 2021 demonstrated a pronounced age effect, and period effects further contributed to increased risks. Metabolic risk factors, including fasting plasma glucose and body mass index, contributed most significantly to the burden. Projections suggest that mortality rates and disability‐adjusted life years are expected to decline by 2036, whereas the incidence is projected to persistently increase.

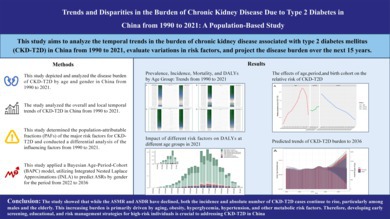

**Summary**

- This study analyzes the burden of chronic kidney disease due to type 2 diabetes (CKD‐T2D) in China from 1990 to 2021 and projects trends through 2036.
- Despite declines in ASMR and ASDR, the incidence and absolute number of cases continue to rise, particularly among older adults and males, driven by aging and metabolic risk factors.
- Early screening and risk management are crucial for mitigating this growing burden.

## 1 Introduction

Diabetic kidney disease (DKD) is one of the most common microvascular complications of type 2 diabetes (T2D) [1]. Clinically, it is primarily characterized by persistent proteinuria and/or a progressive decline in estimated glomerular filtration rate (eGFR) [2]. DKD is the most prevalent form of chronic kidney disease (CKD) worldwide and a leading cause of end‐stage renal disease (ESRD) [3]. Statistics indicate that approximately 20%–40% of patients with diabetes mellitus (DM) are also affected by CKD [4, 5]. Globally, an estimated 20%–30% of ESRD cases are attributed to DKD [6]. DKD exacerbates the risk of renal failure and cardiovascular mortality [7], and leads to an exponential rise in healthcare costs [8], imposing a significant global disease burden. In China, CKD‐T2D has overtaken glomerulonephritis as the leading cause of CKD [9]. However, studies have shown that the awareness of CKD‐T2D among patients with DM in China remains below 20%, and the rate of early treatment is under 50%. As a result, the disease burden of CKD‐T2D continues to be severe in China. Age and hyperglycemia are the primary risk factors for CKD‐T2D [10], while research also identifies obesity, metabolic abnormalities, and unhealthy lifestyles as contributing factors to the development and progression of the disease [11, 12]. However, these time‐related factors often interact, complicating the quantification of each factor's independent contribution in epidemiological analyses. In China, rapid urbanization and economic development have led to significant changes in dietary patterns and a sharp decline in physical activity, resulting in a marked increase in overweight and obesity rates [13]. The fast‐paced lifestyle has further contributed to a sedentary way of living, exacerbating the risk of metabolic disorders. Additionally, the acceleration of population aging, combined with the cumulative effect of diabetes progression over time, has made the elderly population particularly susceptible to DKD [14, 15].

Currently, Chinese patients have limited awareness of the burden of CKD‐T2D, and early diagnosis and treatment remain major challenges in clinical practice. Existing research lacks a clear understanding of the disease burden of CKD‐T2D in China, as well as the trends in its development and the temporal variations in its influencing factors. Although China has introduced policies such as the Healthy China Initiative (2019–2030) [16] and the Medium‐ to Long‐Term Plan for the Prevention and Control of Chronic Diseases in China (2017–2025) [17], which emphasize early screening and management of diabetes and its complications, along with the gradual implementation of standardized and tiered diabetes care at the primary healthcare level, these initiatives still face challenges, including low disease awareness, insufficient early interventions, and uneven distribution of healthcare resources. Therefore, this study utilizes the latest Global Burden of Disease (GBD) data to systematically analyze trends in the burden of CKD‐T2D in China from 1990 to 2021. We will assess the effects of age, period, and cohort, explore the temporal variations in risk factors, and project the disease burden trends for CKD‐T2D over the next 15 years. The study aims to provide precise data‐driven insights to support the optimization of early screening, diagnosis, and prevention strategies for CKD‐T2D in China, thereby informing policy formulation and improving clinical practice.

## 2 Methods

### 2.1 Data Sources

Data on the burden of CKD‐T2D in China from 1990 to 2021 were obtained from the GBD 2021 public database (https://ghdx.healthdata.org/) [18]. The GBD 2021 study employed the latest epidemiological data and refined standardized methodologies to comprehensively evaluate health losses associated with 371 diseases, injuries, and disabilities, as well as 88 risk factors, across 204 countries and territories worldwide [19, 20]. This study focused on individuals diagnosed with CKD‐T2D in China. Estimates of incidence, prevalence, mortality, and DALYs, along with their 95% uncertainty intervals (UIs), were extracted from the GBD 2021 database. Special emphasis was placed on analyzing sex‐ and age‐specific incidence, prevalence, mortality, and DALYs, as well as identifying the risk factors contributing to the burden of CKD‐T2D. Furthermore, the Institutional Review Board (IRB) of the University of Washington waived the requirement for informed consent for obtaining GBD data [1]. This study adhered to the Guidelines for Accurate and Transparent Health Estimates Reporting (GATHER) [21].

### 2.2 Disease Criteria

The disease in this study is CKD‐T2D (ICD‐10 code E11.2–E11.29) [18]. In the GBD study, diabetes is defined as a fasting plasma glucose (FPG) ≥ 7 mmol/L (126 mg/dL), or as individuals who are using insulin or other diabetes medications [22]. CKD‐T2D is defined as CKD due to T2D, characterized by kidney damage that persists for more than 3 months. The primary diagnostic criteria for CKD‐T2D include a urine albumin‐to‐creatinine ratio (UACR) of ≥ 30 mg/g and/or an eGFR of < 60 mL/min/1.73 m^2^ [23, 24].

### 2.3 Descriptive Analysis

To quantify the burden of CKD‐T2D in China, this study conducted a comprehensive evaluation of its prevalence, incidence, mortality, and DALYs. The study visually illustrated and comparatively analyzed the case numbers, crude rates, and age‐standardized rates (ASR) for the prevalence, incidence, mortality, and DALYs of CKD‐T2D patients, stratified by age group and gender, in China from 1990 to 2021.

### 2.4 Trend Analysis

Analyzing temporal trends of a disease is a critical approach to assessing its burden and plays a vital role in guiding precision intervention strategies. This study examines the temporal trends of CKD‐T2D from both local and overall perspectives. The joinpoint regression model (JRM) was employed to analyze the temporal trends in the CKD‐T2D disease burden from 1990 to 2021. This analysis involved calculating the average annual percent change (AAPC) and the annual percent change (APC) for each identified time segment within the model. The JRM fitting and the calculation of AAPC and APC were performed using joinpoint software, version 4.9.1 [25]. The JRM is a method that utilizes segmented regression to analyze the temporal characteristics of disease data distribution, enabling a more detailed assessment of disease trend changes across specific intervals within the overall time frame [26]. The model employs a grid search method to identify all potential joinpoints for segmented functions and applies Monte Carlo permutation tests to optimally determine the number of joinpoints and estimate the corresponding model parameters.

Additionally, this study analyzed trends in the burden of CKD‐T2D by examining the effects of age, period, and birth cohort. First, the potential two‐way interaction effects among age, period, and cohort were investigated (Figures S1 and S2). However, due to the inherent linear dependency among these factors [27], accurately estimating the distinct effects of each component presents a significant challenge. To address this, the study utilized an age‐period‐cohort (APC) model to further analyze the temporal trends in CKD‐T2D incidence and prevalence across the dimensions of age, period, and birth cohort. Based on the Poisson distribution, the APC model applies the intrinsic estimator (IE) method to account for the linear dependency among age, period, and cohort, thereby minimizing potential bias in the results [28, 29]. The basic expression of the model is as follows:

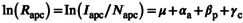

Where ln(*R*_apc_) represents the natural logarithm of the incidence rate; *I*_apc_ represents the number of cases; *N*_apc_ represents the total population surveyed; *μ* represents the total population surveyed; *α*_a_ represents the age effect for the *a*th age group; *β*_p_ represents the period effect for the *p*th period; and *γ*_c_ represents the cohort effect for the *c*th birth cohort. In the APC model applied in this study, the data series were segmented into continuous 5‐year intervals spanning from 1990 to 2021. Data from 1990 to 1991 were excluded from the analysis as they did not constitute a complete 5‐year interval.

Additionally, to control for potential confounding factors that may affect the estimation of disease burden trends, we conducted stratified analyses, including sex‐stratified and age‐stratified modeling, to assess different gender and age groups. This approach helps identify heterogeneity among subpopulations and minimizes the impact of confounding factors on the results. Finally, integrating epidemiological prior knowledge, we systematically interpret the trends in age, period, and cohort effects by considering the natural history of the disease, advancements in medical technology, changes in public health policies, and lifestyle variations across different cohorts. This ensures the validity of the model results and their epidemiological significance.

### 2.5 Risk Factor Attribution Analysis

The GBD 2021 study, grounded in the theoretical framework of comparative risk assessment, uses a counterfactual analysis approach to evaluate and calculate the attributable burden of risk factors. In this study, we analyzed risk factors strongly associated with CKD‐T2D, drawing on clinical practice guidelines and evidence from previous research [12, 30]. To calculate the disease burden attributable to risk factors, the GBD 2021 utilized a rule‐based synthesis of evidence to provide comparable risk quantifications over time and across populations [19]. The study calculates the PAF for each risk factor. The sum of PAFs for these factors may exceed 100%, as many risk factors exert their effects either partially or entirely through one or more other risk factors. In this analysis, DALYs are used to assess the attributable burden of these risk factors [19].

### 2.6 Predictive Analysis

To forecast trends in ASRs of CKD‐T2D beyond 2021, this study applied a Bayesian Age‐Period‐Cohort (BAPC) model, utilizing integrated nested Laplace approximations (INLA) to predict ASRs by gender for the period from 2022 to 2036 [31]. The model assumes that age, period, and cohort effects exert similar influences within adjacent time intervals. By incorporating observed data with prior distributions through Bayesian inference, the model uses a second‐order random walk to smooth these effects, allowing for more accurate predictions of future trends. INLA is used to estimate the marginal posterior distributions, effectively addressing the mixing and convergence issues associated with traditional Markov Chain Monte Carlo sampling methods. The model not only reveals the complex impacts of age, period, and cohort factors on CKD‐T2D but also provides a basis for future trend predictions, thereby supporting the formulation of public health strategies.

All data analysis in this study was conducted using the open‐source software R (version 4.4.1).

## 3 Results

### 3.1 Description and Analysis of the Burden and Overall Trends of CKD‐T2D in China

In 2021, the burden of CKD‐T2D remained substantial, with a total of 20 911 520 cases (95% UI: 19 184 463–22 605 470). Despite the significant increase in the absolute number of cases, the age‐standardized prevalence rate (ASPR) decreased to 1053.92 per 100 000 population (95% UI: 971.11–1139.64). From 1990 to 2021, the overall prevalence of CKD‐T2D exhibited a downward trend, declining by 13% (95% UI: 11%–16%) compared to 1990 (Table 1, Figure S3). However, the incidence of CKD‐T2D continues to rise. In 2021, the number of new CKD‐T2D cases in China was 354 157 (95% UI: 321 265–382 784), with an age‐standardized incidence rate (ASIR) of 16.29 per 100 000 population (95% UI: 14.92–17.53). Between 1990 and 2021, the ASIR increased by 8% (95% UI: 0%–18%), indicating a persistent upward trend in the incidence of CKD‐T2D (Table 1, Figure 3). In 2021, CKD‐T2D was responsible for an estimated 107 652 deaths (95% UI: 84 626–134 047), with an age‐standardized DALY rate (ASDR) of 5.64 per 100 000 population (95% UI: 4.46–7.00). The mortality rate due to CKD‐T2D in China has shown a declining trend, with a 17% decrease (95% UI: 2%–37%) in the age‐standardized mortality rate (ASMR) compared to 1990 (Table 1, Figure S3). In 2021, the number of DALYs attributed to CKD‐T2D in China was 2 537 070 (95% UI: 2 044 338–3 072 897), with an ASDR of 122.15 per 100 000 population (95% UI: 99.62–146.99). Compared to 1990, the DALYs decreased by 22% (95% UI: 6%–37%) in 2021 (Table 1, Figure S3). Overall, from 1990 to 2021, the number of cases and crude rates of incidence, prevalence, mortality, and DALYs for CKD‐T2D in China showed significant increasing trends. However, the ASPR, ASMR, and ASDR exhibited decreasing trends. Notably, the ASIR continued to rise, with males exhibiting a significantly higher ASIR than females (Figure S3).

**TABLE 1:** Number and age‐standardized rate for CKD‐T2D prevalence, incidence, mortality, and disability‐adjusted life years in 1990 and 2021, and the percentage changes from 1990 to 2021 by sex in China.

Measure	1990			2021			Percentage change 1990–2021		
	Male	Female	Total	Male	Female	Total	Male	Female	Total
All‐age cases (*n*)									
Prevalence (95% UI)	5 913 926 (5 344 396, 6 504 759)	5 976 596 (5 412 193, 6 565 190)	11 890 522 (10 790 476, 13 078 708)	10 116 315 (9 283 872, 10 942 400)	10 795 205 (9 866 263, 11 741 118)	20 911 520 (19 184 463, 22 605 470)	0.71 (0.62, 0.8)	0.81 (0.70, 0.90)	0.76 (0.66, 0.85)
Incidence (95% UI)	66 801 (58 778, 74 694)	60 759 (53 787, 68 366)	127 561 (112 718, 142 654)	177 163 (161 643, 191 530)	176 994 (160 794, 191 804)	354 157 (321 265, 382 784)	1.65 (1.45, 1.90)	1.91 (1.63, 2.25)	1.78 (1.55, 2.06)
Deaths (95% UI)	20 352 (15 618, 26 884)	23 186 (18 631, 28 722)	43 537 (35 988, 53 065)	56 959 (41 145, 75 559)	50 693 (37 927, 64 177)	107 652 (84 626, 134 047)	1.80 (0.80, 2.84)	1.19 (0.54, 1.97)	1.47 (0.89, 2.09)
DALYs (95% UI)	587 754 (450 891, 751 005)	643 763 (517 496, 775 929)	1 231 518 (1 022 061, 1 492 725)	1 326 063 (1 025 571, 1 698 910)	1 211 007 (937 561, 1 514 550)	2 537 070 (2 044 338, 3 072 897)	1.26 (0.59, 1.95)	0.88 (0.43, 1.45)	1.06 (0.66, 1.49)
Age‐standardized rate, per 100 000									
Prevalence (95% UI)	1201.87 (1104.76, 1301.29)	1231.54 (1116.32, 1340.19)	1214.76 (1109.34, 1320.7)	1040.72 (958.25, 1124.45)	1069.36 (981.62, 1164.77)	1053.92 (971.11, 1139.64)	−0.13 (−0.16, −0.11)	−0.13 (−0.16, −0.10)	−0.13 (−0.16, −0.11)
Incidence (95% UI)	16.67 (14.77, 18.55)	13.92 (12.4, 15.60)	15.11 (13.45, 16.8)	17.12 (15.73, 18.47)	15.68 (14.34, 16.97)	16.29 (14.92, 17.53)	0.03 (−0.04, 0.11)	0.13 (0.03, 0.25)	0.08 (0.00, 0.18)
Deaths (95% UI)	7.68 (6.05, 10.19)	6.49 (5.24, 8.00)	6.83 (5.74, 8.34)	7.15 (5.28, 9.36)	4.74 (3.52, 6.03)	5.64 (4.46, 7.00)	−0.07 (−0.40, 0.23)	−0.27 (−0.48, −0.03)	−0.17 (−0.37, 0.02)
DALYs (95% UI)	161.80 (128.23, 207.69)	155.30 (125.98, 186.62)	155.94 (131.16, 188.26)	140.79 (109.38, 179.93)	109.35 (84.66, 137.07)	122.15 (99.62, 146.99)	−0.13 (−0.39, 0.13)	−0.30 (−0.46, −0.10)	−0.22 (−0.37, −0.06)

**FIGURE 1:**
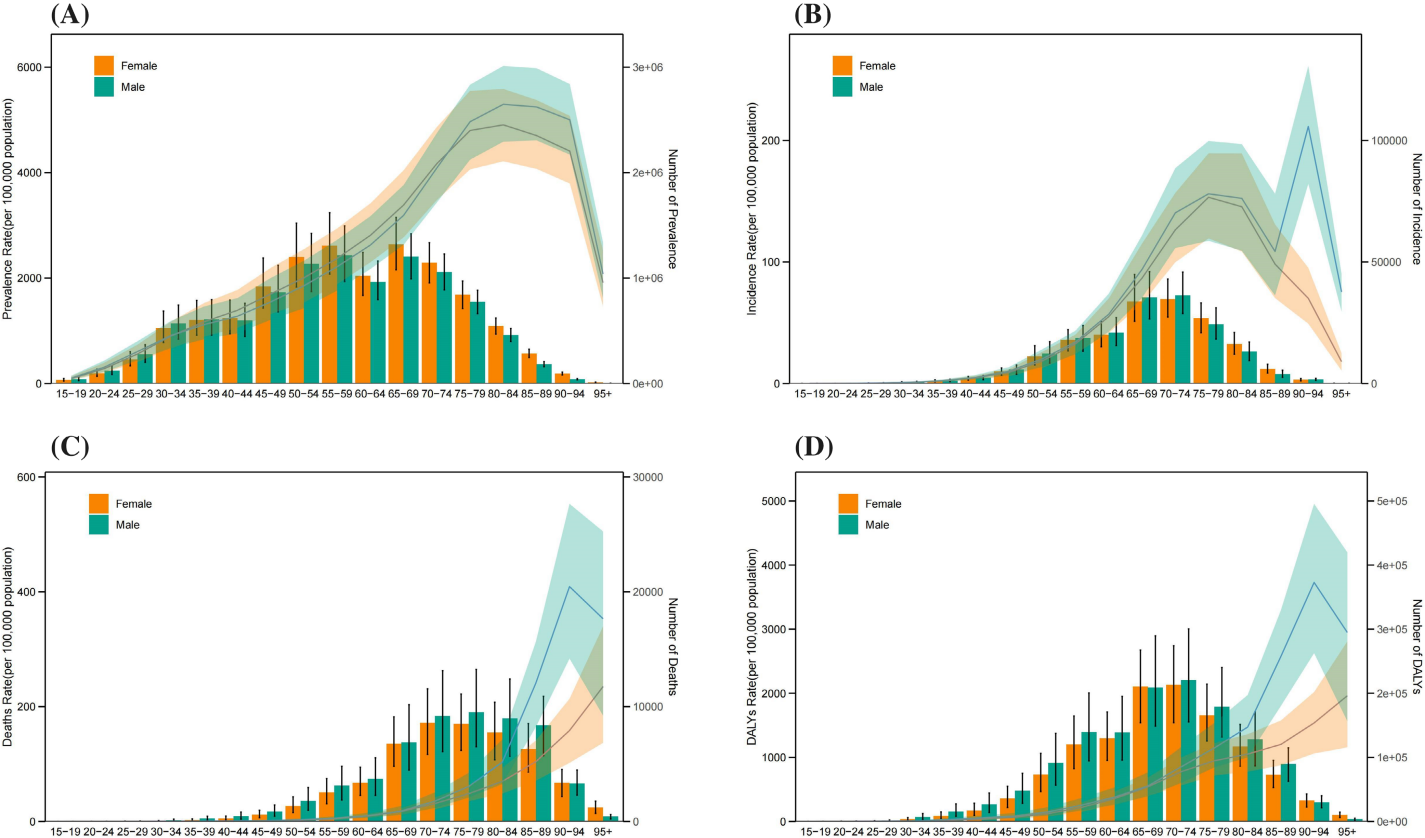
Gender and age structure analysis of the burden of CKD‐T2D in China, 2021. (A) Prevalence; (B) incidence; (C) mortality; (D) DALYs.

This study also analyzed the gender and age distribution of CKD‐T2D patients in China. In 2021, the prevalence of CKD‐T2D peaked in the 80–84 age group, with a slightly higher prevalence observed among males compared to females (Figure 1A). The incidence of CKD‐T2D reached its first peak in the 65–74 age group, followed by a declining trend. However, among males, the incidence reached a second peak in the 90–94 age group, which was significantly higher than that observed in females (Figure 1B). The mortality among CKD‐T2D patients in China gradually increases with age and is significantly higher for males compared to females. Notably, the prevalence of CKD‐T2D in males shows a decline around the age of 90 (Figure 1C). The trend in DALYs follows a similar pattern to that of the death rate (Figure 1D). It is important to note that the number of cases for prevalence, incidence, mortality, and DALYs all peak in the 65–75 age group and then gradually decrease with advancing age. This pattern is likely related to the natural lifespan of patients (Figure 1).

**FIGURE 2:**
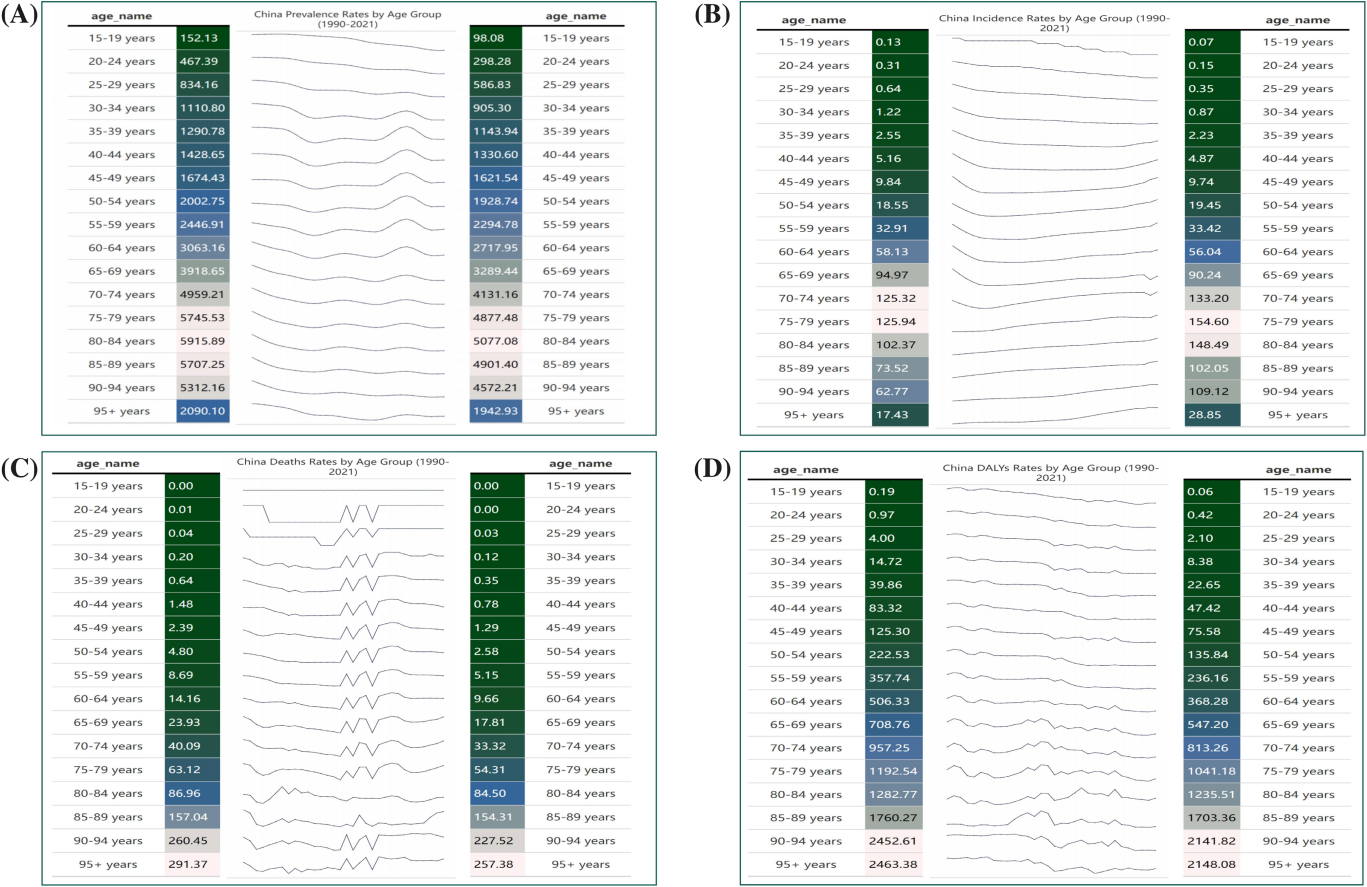
Prevalence, incidence, mortality, and DALYs by age group: trends from 1990 to 2021. (A) Prevalence rates; (B) incidence rates; (C) deaths rates; (D) DALYs rates.

In addition, this study analyzed the annual changes in the prevalence, incidence, mortality, and DALYs of CKD‐T2D patients across different age groups in China from 1990 to 2021. Overall, the prevalence of CKD‐T2D showed a declining trend. However, in the 36–64 age group, the incidence exhibited significant fluctuations (Figure 2A). Unlike the prevalence, the incidence of CKD‐T2D showed an overall increasing trend across most age groups. However, in the 15–34 age group, the incidence has been slowly declining over time. In recent years, the incidence in the 60–74 age group experienced a brief decline, followed by a resurgence (Figure 2B). The mortality for CKD‐T2D in China showed an overall declining trend from 1990 to 2021. However, there were noticeable fluctuations in the death rate during this period, particularly in recent years, where the death rate among individuals aged 65 and older increased significantly (Figure 2C). Similarly, DALYs also exhibited an overall downward trend, with more pronounced fluctuations observed in the 65 and older age group over the years (Figure 2D).

### 3.2 Local Trends in CKD‐T2D Burden Using Joinpoint Regression Analysis

**FIGURE 3:**
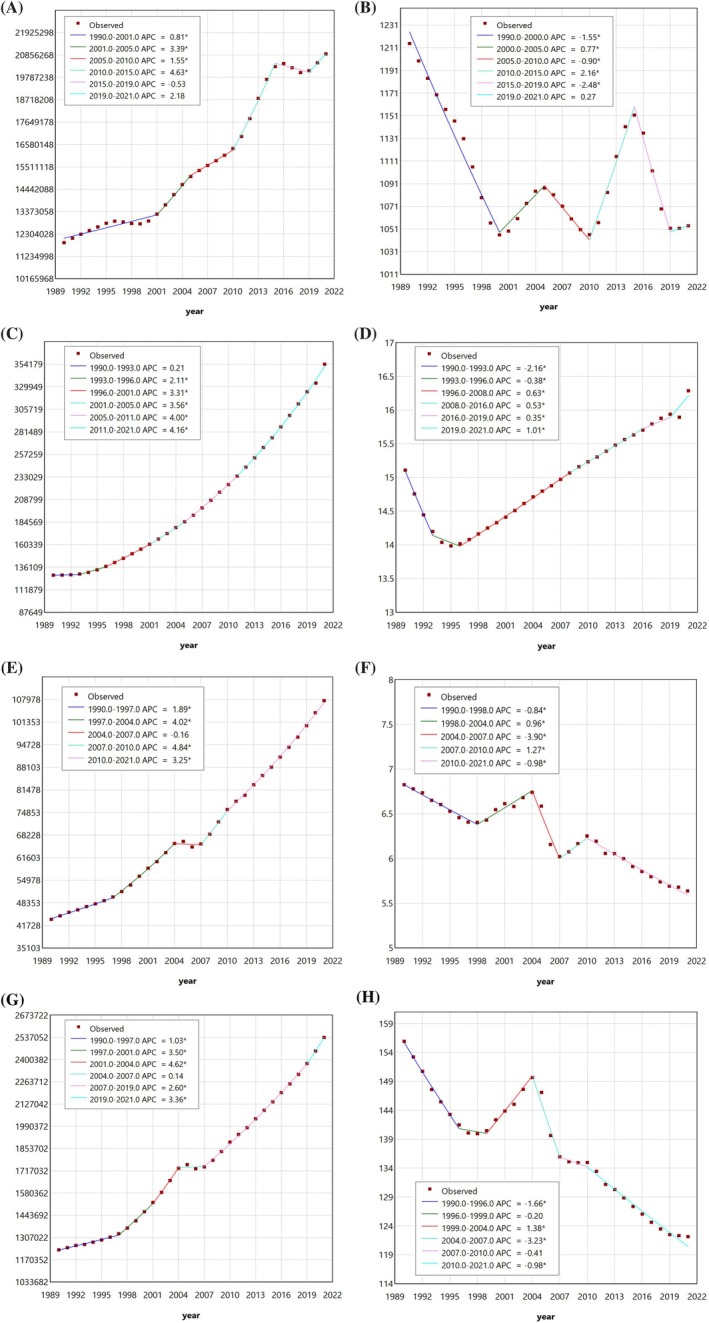
Joinpoint regression analysis of CKD‐T2D burden trends in China. (A) The joinpoint regression analysis on the case number of prevalence; (B) the joinpoint regression analysis on the ASR of prevalence; (C) the joinpoint regression analysis on the case number of incidence; (D) the joinpoint regression analysis on the ASR of incidence; (E) the joinpoint regression analysis on the case number of deaths; (F) the joinpoint regression analysis on the ASR of deaths; (G) the joinpoint regression analysis on the case number of DALYs; (H) the joinpoint regression analysis on the ASR of DALYs; of CKD‐T2D in China.

This study employed joinpoint regression analysis to examine the local trends of the CKD‐T2D burden in China. Between 1990 and 2021, the number of cases for prevalence, incidence, mortality, and DALYs all showed a continuous upward trend. The most rapid growth occurred during 2010–2015, 2011–2021, 2007–2010, and 2001–2004, respectively (Figure 3, Table S1). Compared to 1990, the ASPR of CKD‐T2D significantly decreased. However, between 2000 and 2021, the local trends of ASPR at different joinpoints exhibited significant fluctuations. Specifically, during the periods 2000–2005, 2010–2015, and 2019–2021, ASPR showed an upward trend, with the greatest increase occurring in the 2010–2015 period. Conversely, during the periods 2005–2010 and 2015–2019, there was a notable downward trend, with the most significant decline observed in the 2015–2019 period (Figure 3B, Table S2). The trend of the ASIR showed a pattern of initially decreasing and then increasing. There was a downward trend from 1990 to 1996, followed by three consecutive joinpoints with increasing trends from 1996 to 2021 (Figure 3D, Table S2). The trend for the ASMR overall showed a decreasing trend, with slight and brief increases observed in the periods 1998–2004 and 2007–2010 (Figure 3F, Table S2). As for the ASDR, compared to 1990, it showed an overall decreasing trend across different periods, with the only upward trend occurring between 1999 and 2004 (Figure 3H, Table S2). In summary, between 1990 and 2021, the overall burden of CKD‐T2D in China experienced varying degrees of change.

### 3.3 Age‐Period‐Cohort Analysis on CKD‐T2D Prevalence and Incidence

**FIGURE 4:**
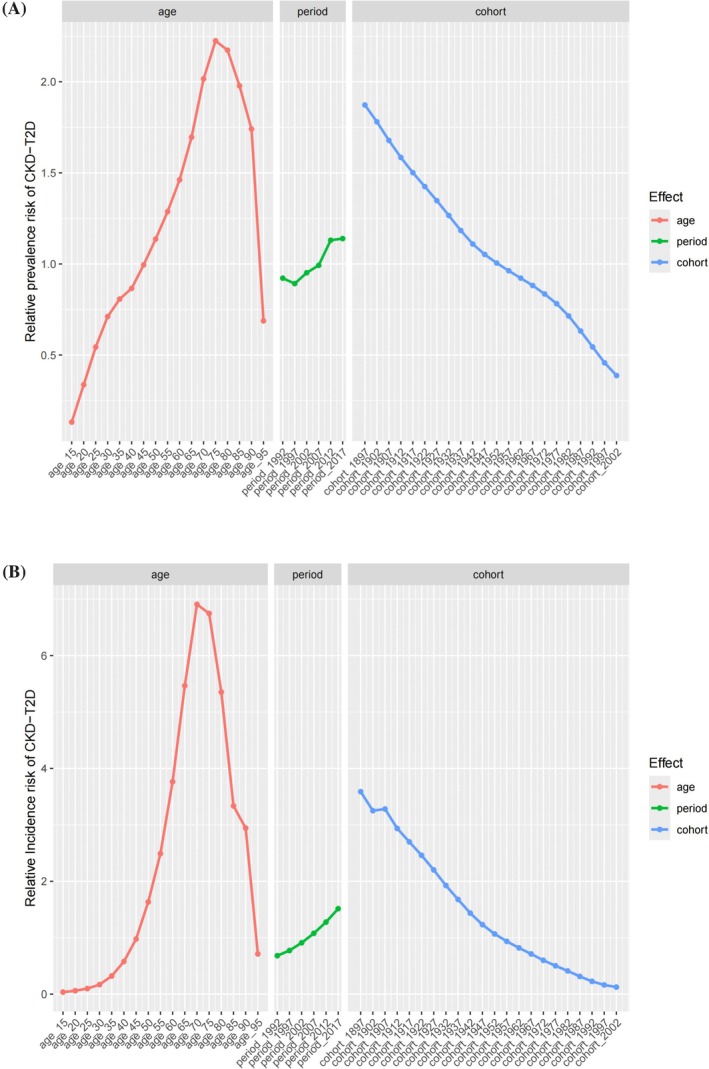
The effects of age, period, and birth cohort on the relative risk of CKD‐T2D. (A) Prevalence and (B) incidence.

This study conducted an APC analysis of the prevalence and incidence of CKD‐T2D in China (Figure 4). Additionally, a sex‐stratified subgroup analysis was conducted (Figures S4 and S5). After controlling for period and cohort effects, the age effect was found to have a significant impact on the risk of CKD‐T2D. Both the relative risks for prevalence and incidence followed a pattern of first increasing and then decreasing, with the highest risks occurring at different age intervals. The highest prevalence risk was observed in the 75–79 age group, while the highest incidence risk was seen in the 70–74 age group (Figure 4, Table S3). After controlling for age and birth cohort effects, the period effect on the risk of CKD‐T2D prevalence and incidence was also found to be significant. Both the prevalence and incidence risks showed an upward trend in the period effect. Specifically, the relative risk (RR) values for the 2017–2021 period group were 1.2 times higher for prevalence and 2.2 times higher for incidence, compared to the 1992–1997 period group. The period from 2017 to 2021 had the highest risk for both the prevalence and incidence of CKD‐T2D (Figure 4, Table S3). After controlling for age and period effects, the birth cohort effect was found to have a significant impact on the risk of CKD‐T2D. The birth cohort effect indicated that earlier‐born cohorts had higher risks of both prevalence and incidence compared to later‐born cohorts, with a continuous decline in risk from the 1987–1901 cohort to the 2002–2006 cohort (Figure 4, Table S3). It is noteworthy that the effects of age, period, and birth cohort on the incidence and prevalence risks of CKD‐T2D, as shown in Figure 4, differ from those presented in Figures S1 and S2. This discrepancy is likely due to the confounding effects of the third factor when analyzing the interactions between any two factors in Figures S1 and S2.

### 3.4 CKD‐T2D‐Related DALYs Attributed to Risk Factors

**FIGURE 5:**
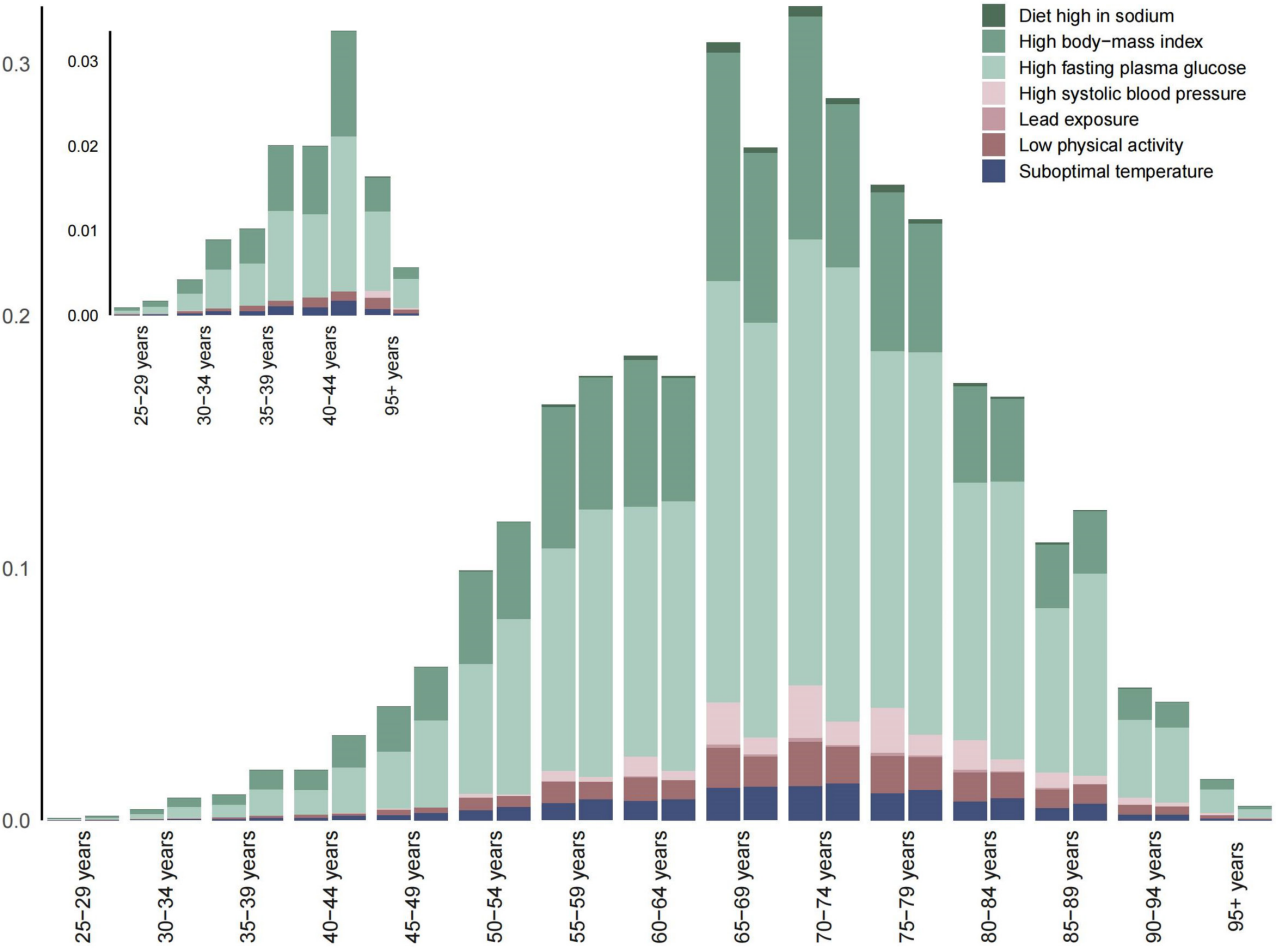
Impact of different risk factors on DALYs at different age groups in 2021.

**FIGURE 6:**
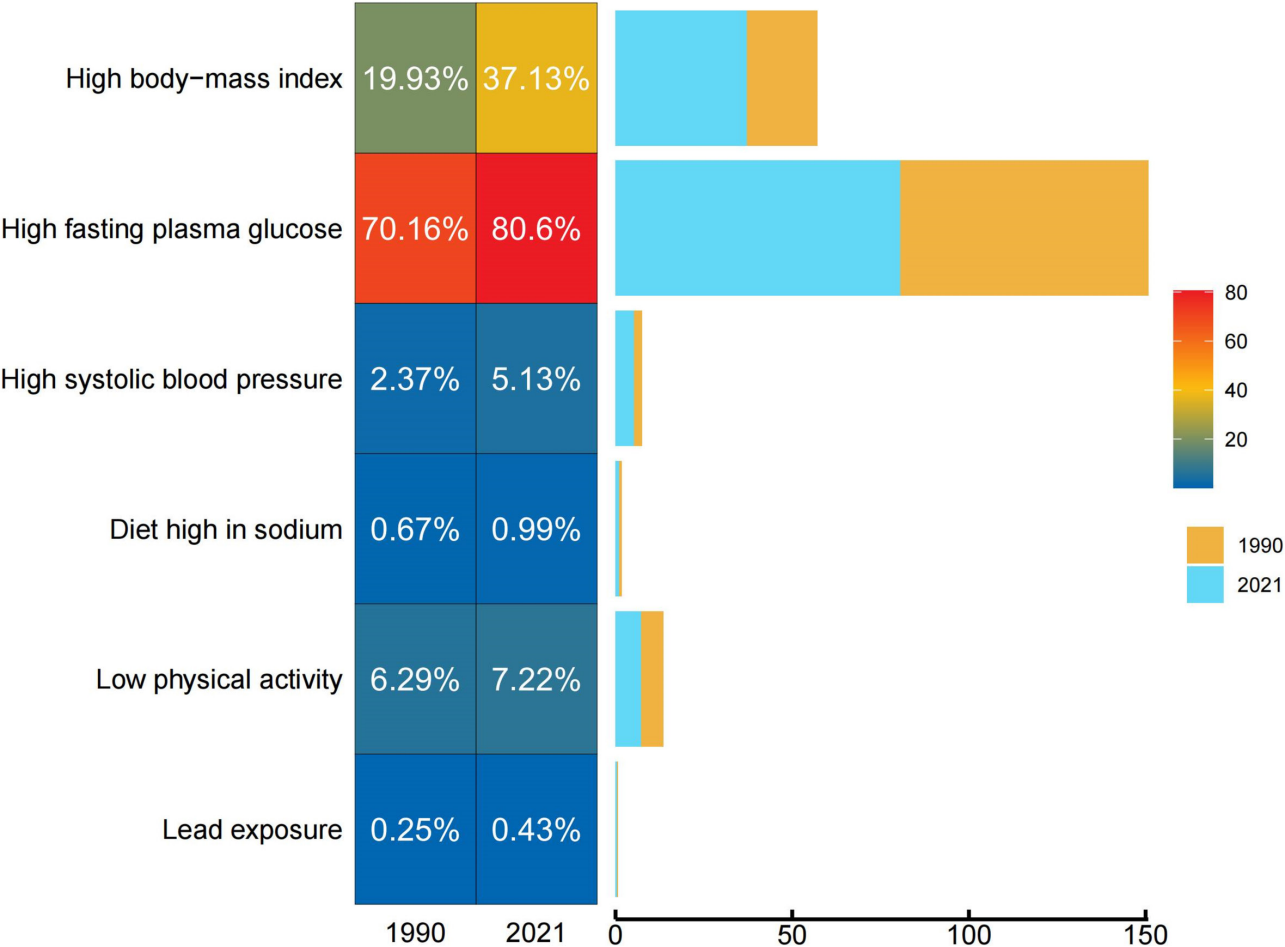
Attributable risk factors for DALYs in 1990 and 2021.

In 2021, various risk factors made significant contributions to the DALYs attributed to CKD‐T2D, affecting all age groups. The proportion of DALYs attributed to individual risk factors for CKD‐T2D varied across different age stages. Overall, in China, high FPG (80.60%) and high body mass index (BMI) (37.13%) were the primary contributors to CKD‐T2D‐related DALYs. Simultaneously, high systolic blood pressure (5.13%), low physical activity (7.22%), and suboptimal temperature (6.33%) also had a significant impact on CKD‐T2D‐related DALYs. Moreover, the influence of factors such as low physical activity and suboptimal temperature on CKD‐T2D‐related DALYs increased with advancing age. Among individuals aged 55 and older, a diet high in sodium also became an important factor affecting DALYs (Figure 5). Additionally, this study conducted a differential analysis of the attributable risk factors between 1990 and 2021 (Figure 6). The results showed that among the seven categories of attributable risks included, all but suboptimal temperature (which decreased from 6.75% to 6.33%) showed a significant upward trend. The study highlights that metabolic factors such as high FPG, high BMI, and high systolic blood pressure have shown the largest increases in risk for CKD‐T2D. Additionally, factors like low physical activity and suboptimal temperature also require continued attention.

### 3.5 Predictive Analysis on CKD‐T2D Burden to 2036

**FIGURE 7:**
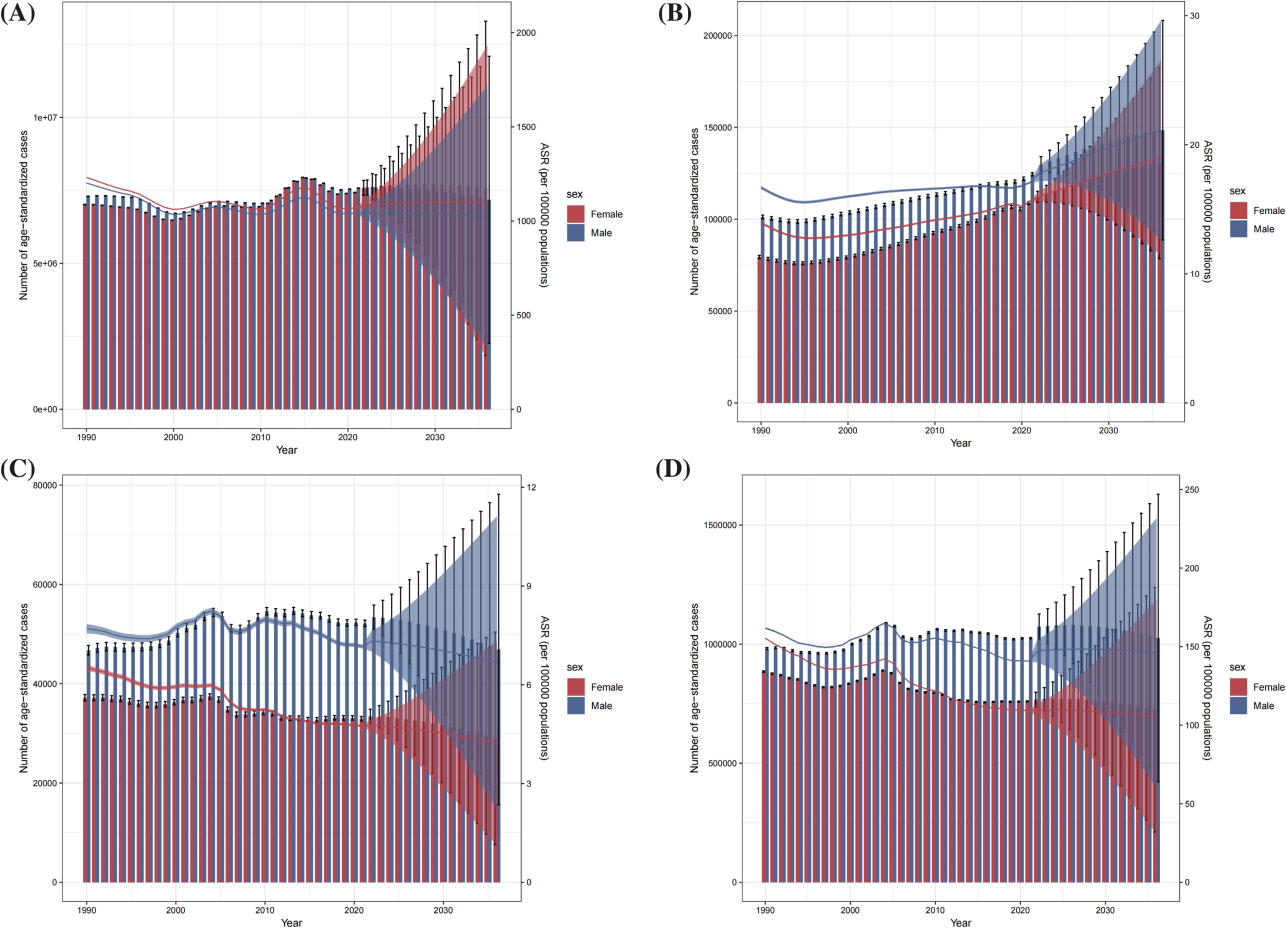
Predicted trends of CKD‐T2D burden to 2036. (A) The predicted case number and ASR of prevalence to 2036; (B) the predicted case number and ASR of incidence to 2036; (C) the predicted case number and ASR of mortality to 2036; (D) the predicted case number and ASR of DALYs to 2036.

This study conducted a predictive analysis on the burden of CKD‐T2D in China for the next 15 years (Figure 7). It is anticipated that the burden of CKD‐T2D in China will undergo significant changes from 2022 to 2036, with different indicators showing varying trends. Overall, it is projected that by 2036, the number of prevalence, mortality, and DALY cases in China will decrease, while the number of new cases will continue to show a clear upward trend. The ASPR for CKD‐T2D is expected to undergo slight changes, remaining largely consistent with the rates in 2021, with the prevalence in females being slightly higher than in males. Specifically, the male ASPR is expected to show a slight downward trend, while the female ASPR will remain relatively stable (Figure 7A). The ASIR of CKD‐T2D is showing a stable upward trend, with the ASIR projected to increase to 20.37 per 100 000 by 2036, and the incidence rate in males is expected to be higher than in females (Figure 7B). The prediction from 2022 to 2036 indicates that the ASMR will continue to decline, reaching a rate of 5.59 per 100 000 people (Figure 7C). The predicted decline in ASDR for CKD‐T2D follows a similar trend to the mortality, but the ASDR for males is still projected to be higher than those for females (Figure 7D). Despite the anticipated control in mortality and DALYs, it is expected that CKD‐T2D will continue to impose a significant disease burden in the future. By 2036, the incidence of new CKD‐T2D cases is still projected to show an upward trend, highlighting the ongoing need for effective prevention and management strategies.

## 4 Discussion

This study conducted a comprehensive analysis of the disease burden of CKD‐T2D in China from 1990 to 2021, revealing a complex landscape of temporal trends in the burden of CKD‐T2D. The results indicate that the burden of CKD‐T2D remains significant in China. In 2021, the number of individuals with CKD‐T2D in China reached 20 911 520 cases, with both the absolute number of cases and the crude prevalence rate continuing to rise over time. This increase in CKD‐T2D burden is largely attributed to changes in population structure, such as an aging population, and the growing prevalence of diabetes, which exacerbates the incidence of CKD [22]. Compared to 1990, the ASIR has increased by 8%, indicating that new cases of CKD‐T2D in China continue to rise, highlighting the need for improved awareness and preventive measures. However, the declining trends in ASIR, ASMR, and DALYs may be attributed to the expansion of healthcare coverage, the implementation of more advanced diabetes and CKD management strategies, and the enhancement of public health awareness and self‐management capabilities. While the strengthening of early screening may have contributed to a higher diagnostic rate, it has also facilitated earlier interventions, helping to slow disease progression and ultimately reduce mortality [32, 33]. Nevertheless, targeted and effective strategies need to be developed for prevention and treatment. Moreover, this study found that the disease burden in males was significantly higher than in females, which may be attributed to the effects of androgens and unhealthy lifestyle factors in males, such as smoking and alcohol consumption [34]. Additionally, the study's findings indicate that the incidence, mortality, and DALYs are higher in individuals aged 65 and older, with significant variations observed across age groups. This also indirectly supports the notion that population aging is a key risk factor for CKD‐T2D.

When the overall trend is divided into multiple sub‐periods, the incidence of CKD‐T2D in China from 1990 to 2021 shows an upward trend, while the mortality and DALYs exhibit fluctuating and downward trends. The results of this study reveal several key turning points in the temporal progression of the disease. In terms of incidence, the incidence of CKD‐T2D in China has shown a sustained upward trend since 1996, which may be linked to the country's economic development and improvements in living standards. Both mortality and DALYs have declined since 2004, likely reflecting improvements in the overall healthcare system in China. Since 2003, China has implemented various urban–rural healthcare policies, which have facilitated the widespread adoption and improvement of medical technologies [35, 36]. Additionally, the study's results reveal a more significant decrease in mortality and DALYs for CKD‐T2D after 2010, which is linked to the Chinese government's provision of free public health services for both urban and rural residents, covering 10 categories and 41 items, including screening and health management for patients with T2D and hypertension [37]. This initiative has indirectly improved the management and control of CKD‐T2D.

Over time, factors such as age, specific historical periods, and birth cohorts can shape human and societal changes. Age has long been recognized as a key factor influencing the development of DKD and CKD [38, 39]. In this study, after adjusting for period and birth cohort effects, the age effect demonstrated a trend of initially increasing and then decreasing relative risks for prevalence and incidence, with the highest risks observed in the age groups of 75–79 and 70–74 years, respectively. The elderly population is heterogeneous and often comorbid with various cardiovascular and metabolic diseases, which can interact and create a vicious cycle that contributes to poor prognosis. Aging has become a significant factor influencing the development of CKD in diabetic patients. The observed decrease in disease risk at advanced ages may be attributed to survival bias, or the possibility that high‐risk individuals do not survive to reach older age brackets [40]. After adjusting for age and birth cohort effects, the period effect significantly influenced the prevalence and incidence of CKD‐T2D. The RR values for the 2017–2021 group were 1.2 times and 2.2 times higher than those for the 1992–1997 group, respectively. This notable increase compared to the earlier period may be attributed to improvements in economic conditions, changes in lifestyle, and the rising incidence of chronic diseases such as cardiovascular disease and hypertension [41]. Furthermore, with advances in medical technology and the widespread dissemination of diabetes education, more individuals may be diagnosed with CKD‐T2D at earlier stages, indirectly contributing to the observed increase in incidence rates. The birth cohort effect indicates that earlier cohorts have higher risks of incidence and prevalence compared to later cohorts. Moreover, this risk has gradually declined with later birth cohorts. Individuals born in earlier cohorts (1901–1947) experienced periods of war, as well as large‐scale natural disasters between 1959 and 1961, during which malnutrition was widespread. Research suggests that nutritional deficiencies during fetal development and early childhood may impair kidney development, thereby increasing the risk of CKD‐T2D in adulthood. Studies have shown that malnutrition is closely linked to changes in renal tissue [42]. Moreover, malnutrition has been shown to increase the risk of cardiovascular and all‐cause mortality [43]. In contrast, individuals born in the 1980s and later grew up during a period of rapid economic development in China, characterized by significant improvements in living standards and nutritional status, as well as the gradual establishment of a chronic disease management system, which has contributed to a reduced risk of CKD‐T2D. Additionally, individuals from earlier birth cohorts had limited access to healthcare resources during their developmental years, with inadequate management of underlying diseases, potentially exacerbating long‐term health risks in adulthood. Although the risk of CKD‐T2D is lower among more recent birth cohorts, modern lifestyle changes, such as increased sedentary behavior, high‐fat diets, and rising adolescent obesity rates may influence future disease burden. This underscores the long‐term impact of birth cohorts on population health and highlights the need for targeted CKD‐T2D prevention and management strategies tailored to different cohorts in the future.

Furthermore, interactions exist among age, period, and cohort effects. Population aging amplifies the period effect, as increased life expectancy among individuals with diabetes leads to a higher prevalence of the disease among older populations. At the same time, medical advancements may reduce the disease burden in certain age groups. The cohort effect is also closely linked to age. Individuals from earlier birth cohorts, who experienced childhood malnutrition and limited healthcare access, face a higher risk of CKD‐T2D in old age. In contrast, while later birth cohorts have a lower initial risk, the persistence of unhealthy lifestyles may increase the diabetes burden at younger ages. Additionally, there is an interaction between period and cohort effects. Earlier cohorts have benefited from advancements in modern medicine and public health policies, leading to a reduction in disease risk. Meanwhile, although later birth cohorts have grown up in a more favorable living environment, they may face new health challenges due to sedentary behavior, unhealthy diets, and other lifestyle‐related risk factors. These complex interactions further shape the long‐term epidemiological trends of CKD‐T2D.

Identifying risk factors is essential for the prevention of CKD‐T2D, and addressing controllable risk factors is a key strategy for reducing the disease burden, which is critical for the development of public health policies. The results of this study show that high FPG (80.60%) and high BMI are the primary contributors to CKD‐T2D‐related DALYs in China. Additionally, high systolic blood pressure (5.13%), low physical activity (7.22%), and suboptimal temperature (6.33%) also significantly impact CKD‐T2D‐related DALYs. Patients with T2D and resulting CKD are at increased risk of premature morbidity and mortality, primarily due to cardiovascular events. Poor control of hypertension and hyperglycemia are common modifiable risk factors [10, 44]. Additionally, obesity is recognized as an independent risk factor for the development of kidney disease, with increased visceral fat often associated with the progression of renal damage [45, 46]. Additionally, studies have shown that low physical activity may contribute to the development of microalbuminuria in patients with T2D, potentially through mechanisms such as sympathetic nervous system activation and reduced baroreflex sensitivity [47]. The intensity of physical activity may be a key determinant in the onset and progression of CKD‐T2D. Renin‐angiotensin system (RAS) blockade is considered crucial in slowing the progression of CKD in diabetic patients. Studies have found that dietary salt restriction may enhance the effects of RAS blockade in T2D patients, potentially helping to control CKD progression [48]. Additionally, suboptimal temperature and occupational exposures can also impact renal function [49, 50]. Therefore, environmental factors should also receive special attention.

Predicting disease trends is a critical component of prevention and control efforts. It is anticipated that by 2036, the mortality, and DALYs in China will decline, while the number of new cases is expected to continue rising. Although some progress has been made in controlling mortality and DALYs, the incidence of CKD‐T2D is expected to continue rising steadily in the future. China's paradox of being able to “control mortality easily but facing challenges in disease prevention” is not unique; its position in the international context further underscores the complexity and urgency of these challenges. From a global perspective, the evolution of CKD‐T2D disease burden in China exhibits an “intermediate state” characteristic. Compared with other developing countries, China's declining trends in ASDR and DALYs rates are significantly more pronounced than those observed in India (where diabetes‐related CKD mortality increased by 53% from 1990 to 2016) [51], Pakistan (where the DALY rate rose by 52.7% from 1990 to 2019) [52], and Mexico (where CKD‐T2D‐related mortality among women surged by 348%) [53]. These trends reflect China's progress in chronic disease management and improved accessibility to healthcare resources. However, similar to high‐income countries such as the United States, where 36% of diabetes patients develop kidney disease [54], China is also facing a continuous rise in incidence, with the total number of cases exceeding 20 million in 2021, and the aging population playing a particularly significant role. On a global scale, metabolic risk factors and population aging remain the primary driving forces behind CKD‐T2D burden. Nevertheless, developing and high‐income countries exhibit distinct characteristics: in developing nations, limited healthcare resources and low health awareness contribute to an increasing disease burden, whereas in high‐income countries, the challenge lies in the high prevalence of lifestyle‐related diseases and the ongoing burden of managing complications. This indicates that China's efforts in CKD‐T2D diagnosis, treatment, and management are beginning to show results. However, the persistently high incidence underscores the ongoing challenges of low awareness and insufficient understanding of CKD‐T2D in the country. As a result, the disease burden remains substantial.

Therefore, a comprehensive strategy is urgently needed to support the prevention and management of CKD‐T2D, aiming to mitigate its public health impact. This includes strengthening early screening and management by establishing an early warning system for CKD‐T2D, with a particular focus on high‐risk populations, and promoting early diagnosis and intervention in primary healthcare settings. Additionally, optimizing the chronic disease management system by expanding medical insurance coverage and reducing the financial burden on patients is crucial. It is also essential to encourage a healthy lifestyle by promoting regular physical activity, reducing sedentary behavior, adopting a balanced diet, and raising awareness of disease prevention. Furthermore, personalized health management plans should be developed for high‐risk individuals to help alleviate the burden of CKD‐T2D and minimize its adverse effects.

Analyzing the burden of CKD‐T2D and its risk factors in China using GBD data is highly valuable for informing treatment, prevention, and management strategies. However, there are certain limitations to consider. First, potential biases may exist in data collection and modeling. Differences in representativeness and consistency across data sources may exist, particularly in resource‐limited remote areas where data may be insufficient or delayed. For example, incomplete death registration or low disease surveillance coverage could lead to either underestimation or overestimation of the disease burden. Additionally, modeling methods may introduce uncertainties, potentially influencing the accuracy of trend projections. Second, changes in diagnostic practices may affect trend interpretations. In recent years, increased screening rates for diabetes and CKD have led to more patients being diagnosed at earlier stages. Consequently, the observed rise in incidence may partially reflect improved detection rather than a true increase in disease burden. Furthermore, evolving diagnostic criteria for CKD and T2D over time may pose challenges to data comparability across different periods. Finally, some key risk factors have not been fully captured, such as genetic predisposition, mental health conditions, and patient adherence to treatment. These factors may influence CKD‐T2D progression, yet due to data limitations, this study is unable to comprehensively assess their impact. Additionally, regional disparities within China and socioeconomic factors may not be fully reflected, limiting the depth of analysis. Moreover, GBD data primarily reveal associations rather than causation, making it necessary to integrate country‐specific and localized data for further validation to ensure applicability in China's healthcare context.

## 5 Conclusion

This study, based on disease burden data for CKD‐T2D in China from 1990 to 2021, reveals the complex trends in prevalence, incidence, and associated burden, and provides predictions for the disease trends over the next 15 years in China. Although mortality and DALYs have decreased, the incidence and absolute number of cases continue to rise, particularly among males and the elderly, reflecting the impacts of population aging and unhealthy lifestyle choices. Key risk factors, including high FPG, high BMI, high systolic blood pressure, and low physical activity, underscore the need for strengthened early screening, public education, and risk management strategies. The findings of this study also indirectly highlight the importance of optimizing healthcare services and improving the environment to comprehensively reduce the disease burden of CKD‐T2D in China.

## Author Contributions

Juan Jin and Qiang He designed the study. Yifei Wang, Shiya Gu, and Zhiyong Xu extracted and analyzed the GBD data. Yifei Wang, Shiya Gu, Zhixuan Xie, Zhiyong Xu, and Yexiang Chen contributed to the statistical analysis and interpretation of data. Yifei Wang and Shiya Gu drafted the manuscript. Zhixuan Xie, Yexiang Chen, and Wenfang He revised the manuscript. Yifei Wang, Juan Jin, and Qiang He accessed and verified the underlying data. The final version was approved by all authors.

## Conflicts of Interest

The authors declare no conflicts of interest.

## Supporting information


Data S1.

## Data Availability

GBD study 2021 data resources were available online from the Global Health Data Exchange (GHDx) query tool (http://ghdx.healthdata.org/gbd‐results‐tool).
