# Supplementary material for: Trends and Disparities in the Burden of Chronic Kidney Disease due to Type 2 Diabetes in China From 1990 to 2021: A Population‐Based Study
Source: J Diabetes. 2025 Apr 23;17(4):e70084. doi: 10.1111/1753-0407.70084 (PMC12015641; doi:10.1111/1753-0407.70084)
Supplement: Supplementary file 1 — Data S1. [file JDB-17-e70084-s001.docx]

**Supplementary Material**

**Contents**

**Figure S1……………………………………………………………………………………. 2**

**Figure S2……………………………………………………………………………………. 3**

**Figure S3……………………………………………………………………………………. 4**

**Figure S4……………………………………………………………………………………. 5**

**Figure S5……………………………………………………………………………………. 6**

**Table S1……………………………………………………………………………………... 7**

**Table S2……………………………………………………………………………………... 8**

**Table S3……………………………………………………………………………………... 9**


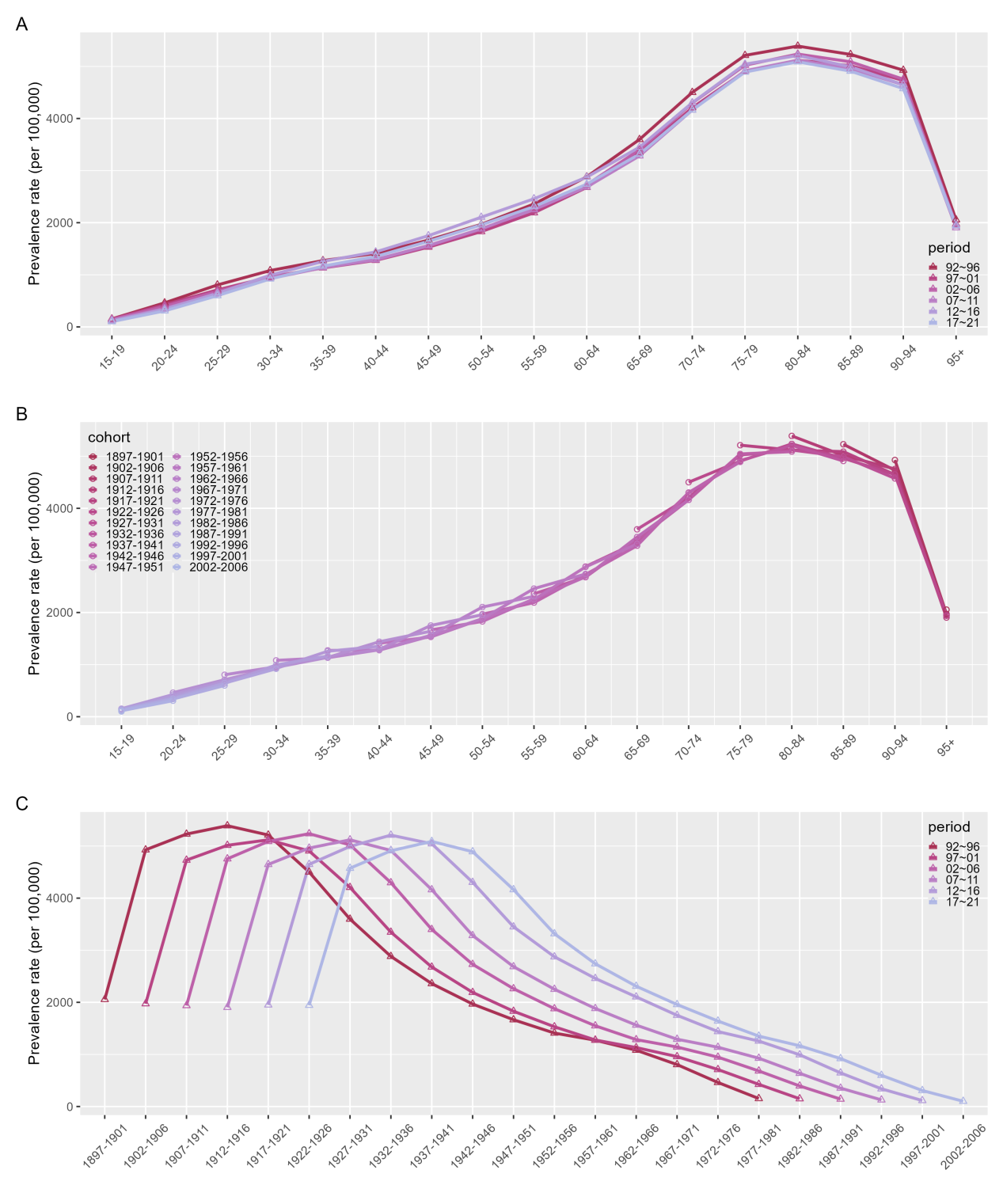


**Figure S1.** The role of interaction between two factors including age-period (A), and cohort-period (B), age-cohort (C) on the Prevalence of CKD-T2D.


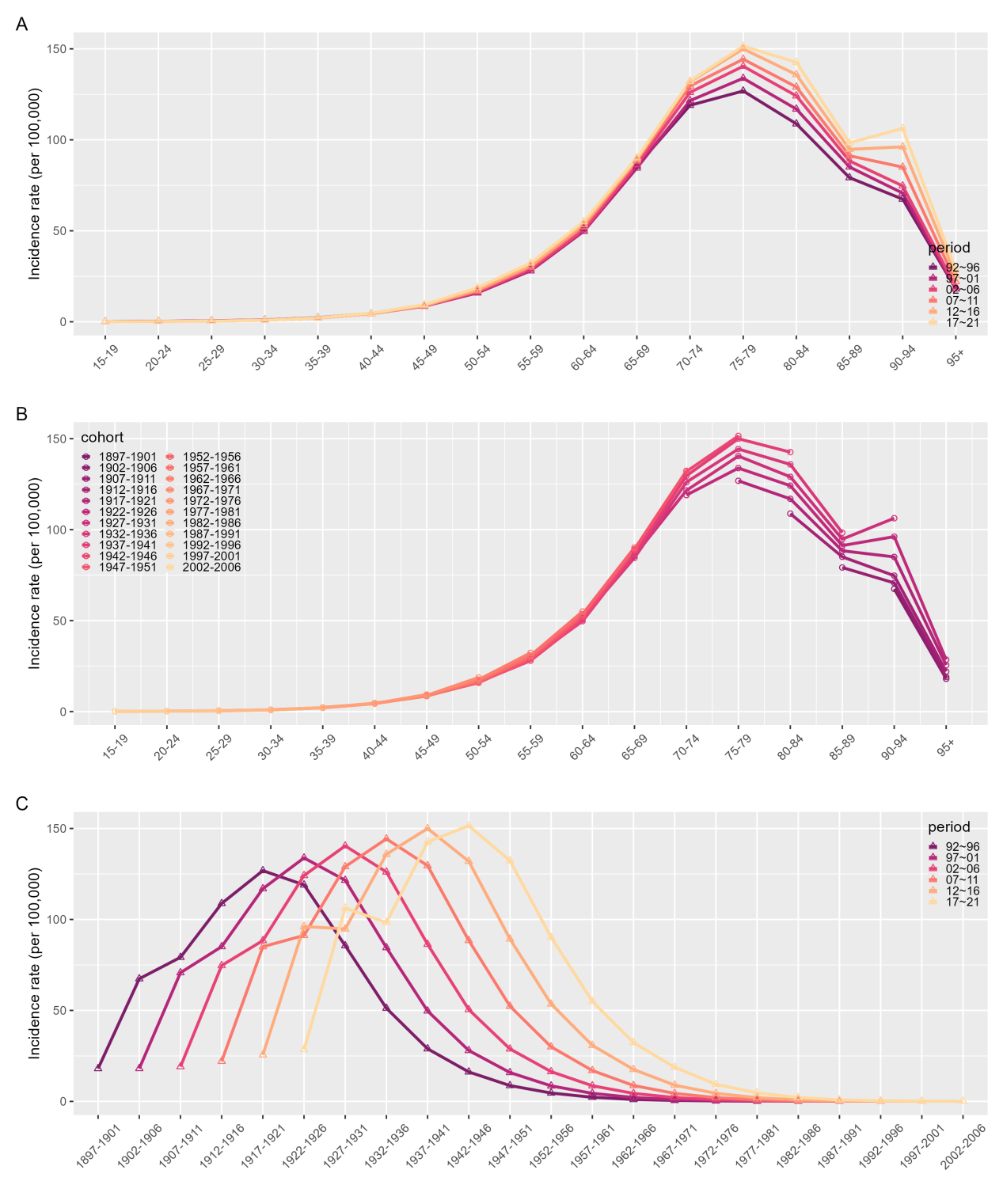


**Figure S2.** The role of interaction between two factors including age-period (A), and cohort-period (B), age-cohort (C) on the Incidence of CKD-T2D


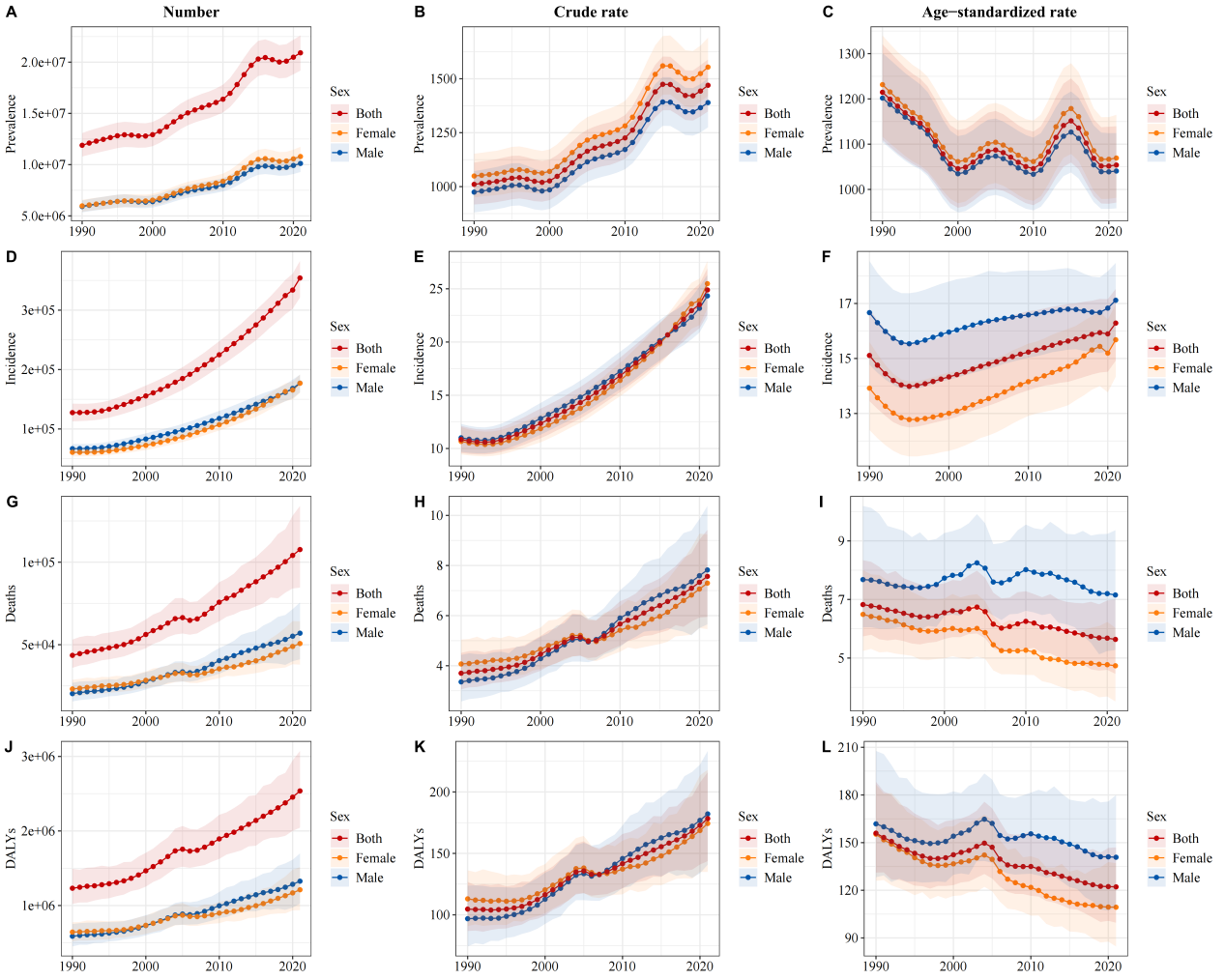


**Figure S3.** The trends of case number, crude rate and age-standardized rate of prevalence, incidence, deaths and DALYs of CKD-T2D from 1990 to 2021.


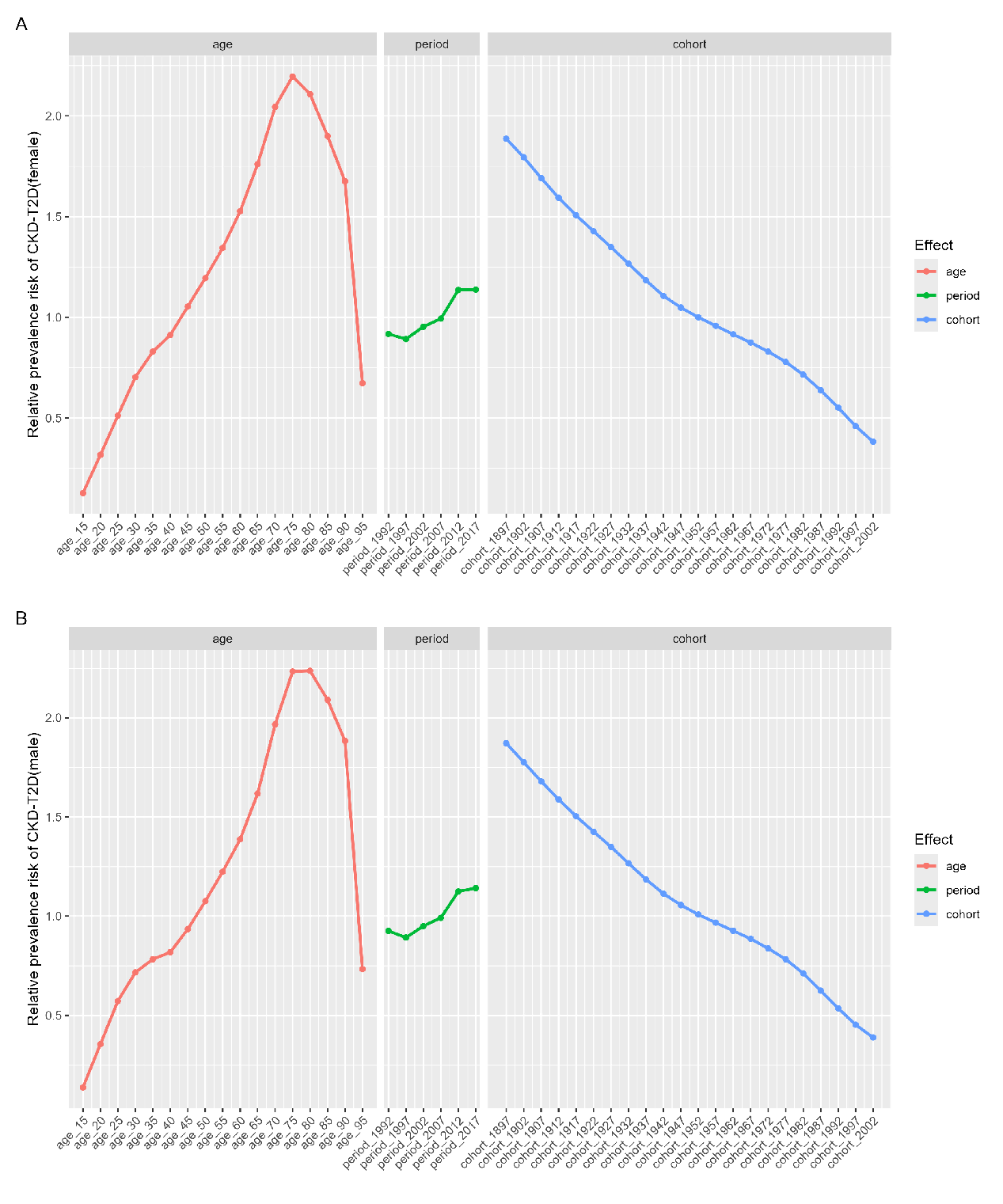


**Figure S4**.The effects of age,period,and birth cohort on the relative risk of CKD-T2D prevalence stratified by sexes.


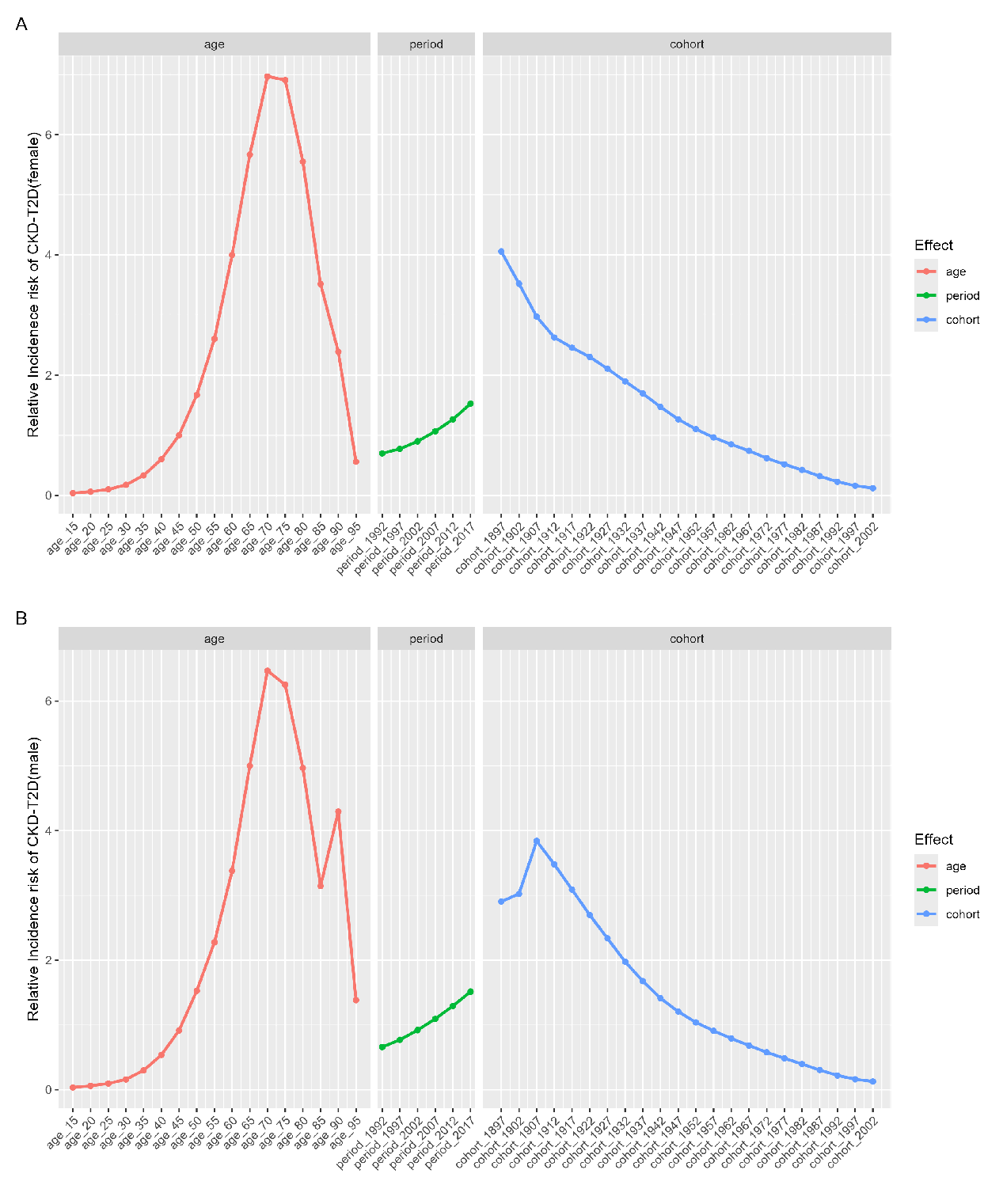


**Figure S5.**The effects of age, period, and birth cohort on the relative risk of CKD-T2D incidence stratified by sexes.

**Table S1** The APC and AAPC in case number of CKD-T2D prevalence, incidence, deaths and DALYs for both sexes from 1990 to 2021

| **Prevalence** | | | **Incidence** | | | **Deaths** | | | **DALYs** | | |
| --- | --- | --- | --- | --- | --- | --- | --- | --- | --- | --- | --- |
| **Range** | **APC (95% CI)** | ***P*** | **Range** | **APC (95% CI)** | ***P*** | **Range** | **APC (95% CI)** | ***P*** | **Range** | **APC (95% CI)** | ***P*** |
| 1990-2001 | 0.81  (0.59 - 1.03) | <0.01 | 1990-1993 | 0.21  (-0.65 - 1.08) | 0.61 | 1990-1997 | 1.89  (1.71 - 2.06) | <0.01 | 1990-1997 | 1.03  (0.91 - 1.15) | <0.01 |
| 2001-2005 | 3.39 (1.83 - 4.98) | <0.01 | 1993-1996 | 2.11  (0.39 - 3.86) | 0.02 | 1997-2004 | 4.02  (3.82 - 4.22) | <0.01 | 1997-2001 | 3.50  (3.07 - 3.93) | <0.01 |
| 2005-2010 | 1.55  (0.63 - 2.48) | <0.01 | 1996-2001 | 3.31  (2.79 - 3.83) | <0.01 | 2004-2007 | -0.16  (-1.22 - 0.91) | 0.76 | 2001-2004 | 4.62  (3.82 - 5.43) | 0.70 |
| 2010-2015 | 4.63  (3.75 - 5.52) | <0.01 | 2001-2005 | 3.56  (2.79 - 4.33) | <0.01 | 2007-2010 | 4.84  (3.77 - 5.93) | <0.01 | 2004-2007 | 0.14  (-0.61 - 0.89) | <0.01 |
| 2015-2019 | -0.53  (-1.79 - 0.75) | 0.39 | 2005-2011 | 4.00  (3.69 - 4.32) | <0.01 | 2010-2021 | 3.25  (3.18 - 3.31) | <0.01 | 2007-2019 | 2.60  (2.56 - 2.65) | <0.01 |
| 2019-2021 | 2.18  (-0.36 - 4.78) | 0.09 | 2011-2021 | 4.16  (4.07 - 4.25) | <0.01 |  |  |  | 2019-2021 | 3.36  (2.72 - 4.01) | <0.01 |
|  | | | | | | | | | | | |
| **Range** | **AAPC (95% CI)** | ***P*** | **Range** | **AAPC (95% CI)** | ***P*** | **Range** | **AAPC (95% CI)** | ***P*** | **Range** | **AAPC (95% CI)** | ***P*** |
| 1990-2021 | 1.78  (1.43 - 2.13) | <0.01 | 1990-2021 | 3.23  (3.11 - 3.55) | <0.01 | 1990-2021 | 2.93  (2.78 - 3.08) | <0.01 | 1990-2021 | 2.36  (2.24 - 2.48) | <0.01 |

**Table S2** The APC and AAPC in ASR of CKD-T2D prevalence, incidence, deaths and DALYs for both sexes from 1990 to 2021

| **Prevalence** | | | **Incidence** | | | **Deaths** | | | **DALYs** | | |
| --- | --- | --- | --- | --- | --- | --- | --- | --- | --- | --- | --- |
| **Range** | **APC (95% CI)** | ***P*** | **Range** | **APC (95% CI)** | ***P*** | **Range** | **APC (95% CI)** | ***P*** | **Range** | **APC (95% CI)** | ***P*** |
| 1990-2000 | -1.55  (-1.68 , -1.41) | <0.01 | 1990-1993 | -2.16  (-2.25 , -2.06) | <0.01 | 1990-1998 | -0.84  (-0.99 , -0.68) | <0.01 | 1990-1996 | -1.66  (-1.90 , -1.43) | <0.01 |
| 2000-2005 | 0.77  (0.24 , 1.31) | 0.01 | 1993-1996 | -0.38  (-0.57 , -0.18) | <0.01 | 1998-2004 | 0.96  (0.69 - 1.24) | <0.01 | 1996-1999 | -0.20  (-1.40 , 1.02) | 0.74 |
| 2005-2010 | -0.90  (-1.41 , -0.38) | <0.01 | 1996-2008 | 0.63  (0.62 , 0.64) | <0.01 | 2004-2007 | -3.90  (-5.12 , -2.68) | <0.01 | 1999-2004 | 1.38  (1.00 - 1.77) | <0.01 |
| 2010-2015 | 2.16  (1.63 , 2.70) | <0.01 | 2008-2016 | 0.53  (0.51 - 0.55) | <0.01 | 2007-2010 | 1.27  (0.01 , 2.54) | 0.05 | 2004-2007 | -3.23  (-4.42 , -2.02) | <0.01 |
| 2015-2019 | -2.48  (-3.29 ,-1.65) | <0.01 | 2016-2019 | 0.35  (0.20 - 0.50) | <0.01 | 2010-2021 | -0.98  (-1.08 , -0.88) | <0.01 | 2007-2010 | -0.41  (-1.56 , 0.74) | 0.46 |
| 2019-2021 | 0.27  (-1.41 , 1.98) | 0.74 | 2019-2021 | 1.01  (0.86, 1.15) | <0.01 |  |  |  | 2010-2021 | -0.98  (-1.07 , -0.89) | <0.01 |
|  | | | | | | | | | | | |
| **Range** | **AAPC (95% CI)** | ***P*** | **Range** | **AAPC (95% CI)** | ***P*** | **Range** | **AAPC (95% CI)** | ***P*** | **Range** | **AAPC (95% CI)** | ***P*** |
| 1990-2021 | -0.48  (-0.68 , -0.29) | <0.01 | 1990-2021 | 0.22  (0.17 , 0.27) | <0.01 | 1990-2021 | -0.64  (-0.81 , -0.47) | <0.01 | 1990-2021 | -0.83  (-1.02 , -0.63) | <0.01 |

**Table S3** RRs of CKD-T2D prevalence and incidence for both sexes due to age, period, and birth cohort effects.

| **Factor** | **Prevalence** | | **Incidence** | |
| --- | --- | --- | --- | --- |
|  | **RR (95% CI)** | **P** | **RR(95%CI)** | **P** |
| **Age (years)** | | | | |
| 15-19 | 0.1318(0.1314, 0.1323) | <0.01 | 0.0365(0.0343, 0.0389) | <0.01 |
| 20-24 | 0.3369(0.3360, 0.3379) | <0.01 | 0.0612(0.0585, 0.0641) | <0.01 |
| 25-29 | 0.5438(0.5424, 0.5451) | <0.01 | 0.0992(0.0957, 0.1028) | <0.01 |
| 30-34 | 0.7113(0.7099, 0.7128) | <0.01 | 0.1693(0.1645, 0.1742) | <0.01 |
| 35-39 | 0.8073(0.8059, 0.8086) | <0.01 | 0.3220(0.3148, 0.3294) | <0.01 |
| 40-44 | 0.8660(0.8649, 0.8672) | <0.01 | 0.5783(0.5681, 0.5887) | <0.01 |
| 45-49 | 0.9948(0.9938, 0.9958) | <0.01 | 0.9767(0.9634, 0.9902) | 0.001 |
| 50-54 | 1.1365(1.1356, 1.1374) | <0.01 | 1.6345(1.6174, 1.6517) | <0.01 |
| 55-59 | 1.2874(1.2864, 1.2884) | <0.01 | 2.4910(2.4691, 2.5131) | <0.01 |
| 60-64 | 1.4617(1.4603, 1.4630) | <0.01 | 3.7652(3.7302, 3.8006) | <0.01 |
| 65-69 | 1.6962(1.6942, 1.6983) | <0.01 | 5.4656(5.4015, 5.5305) | <0.01 |
| 70-74 | 2.0158(2.0128, 2.0190) | <0.01 | 6.9078(6.8030, 7.0141) | <0.01 |
| 75-79 | 2.2247(2.2205, 2.2290) | <0.01 | 6.7461(6.6173, 6.8774) | <0.01 |
| 80-84 | 2.1735(2.1685, 2.1786) | <0.01 | 5.3537(5.2289, 5.4816) | <0.01 |
| 85-89 | 1.9777(1.9721, 1.9833) | <0.01 | 3.3364(3.2425, 3.4331) | <0.01 |
| 90-94 | 1.7404(1.7341, 1.7467) | <0.01 | 2.9425(2.8417, 3.0468) | <0.01 |
| 95+ | 0.6876(0.6819, 0.6934) | <0.01 | 0.7134(0.6610, 0.7700) | <0.01 |
| **Period** | | | | |
| 1992 | 0.9219(0.9209, 0.9229) | <0.01 | 0.6815(0.6735, 0.6896) | <0.01 |
| 1997 | 0.8920(0.8914, 0.8927) | <0.01 | 0.7734(0.7677, 0.7793) | <0.01 |
| 2002 | 0.9517(0.9513, 0.9521) | <0.01 | 0.9103(0.9068, 0.9137) | <0.01 |
| 2007 | 0.9923(0.9919, 0.9927) | <0.01 | 1.0776(1.0738, 1.0815) | <0.01 |
| 2012 | 1.1303(1.1295, 1.1311) | <0.01 | 1.2767(1.2675, 1.2860) | <0.01 |
| 2017 | 1.1392(1.1380, 1.1404) | <0.01 | 1.5148(1.4974, 1.5324) | <0.01 |
| **Birth cohort** | | | | |
| 1987-1901 | 1.8725(1.8025, 1.9453) | <0.01 | 3.5880(2.3957, 5.3736) | <0.01 |
| 1902-1906 | 1.7801(1.7623, 1.7981) | <0.01 | 3.2512(2.9595, 3.5717) | <0.01 |
| 1907-1911 | 1.6784(1.6677, 1.6892) | <0.01 | 3.2814(3.0769, 3.4994) | <0.01 |
| 1912-1916 | 1.5845(1.5760, 1.5931) | <0.01 | 2.9369(2.7779, 3.1050) | <0.01 |
| 1917-1921 | 1.5010(1.4938, 1.5083) | <0.01 | 2.6980(2.5652, 2.8378) | <0.01 |
| 1922-1926 | 1.4247(1.4185, 1.4309) | <0.01 | 2.4603(2.3496, 2.5763) | <0.01 |
| 1927-1931 | 1.3476(1.3423, 1.3529) | <0.01 | 2.2019(2.1116, 2.2960) | <0.01 |
| 1932-1936 | 1.2660(1.2615, 1.2705) | <0.01 | 1.9261(1.8546, 2.0003) | <0.01 |
| 1937-1941 | 1.1838(1.1801, 1.1875) | <0.01 | 1.6780(1.6221, 1.7359) | <0.01 |
| 1942-1946 | 1.1095(1.1064, 1.1125) | <0.01 | 1.4359(1.3932, 1.4800) | <0.01 |
| 1947-1951 | 1.0521(1.0497, 1.0546) | <0.01 | 1.2319(1.1993, 1.2653) | <0.01 |
| 1952-1956 | 1.0049(1.0029, 1.0069) | <0.01 | 1.0676(1.0425, 1.0932) | <0.01 |
| 1957-1961 | 0.9629(0.9614, 0.9645) | <0.01 | 0.9359(0.9162, 0.9561) | <0.01 |
| 1962-1966 | 0.9221(0.9210, 0.9233) | <0.01 | 0.8198(0.8038, 0.8361) | <0.01 |
| 1967-1971 | 0.8817(0.8809, 0.8825) | <0.01 | 0.7125(0.6991, 0.7262) | <0.01 |
| 1972-1976 | 0.8351(0.8345, 0.8357) | <0.01 | 0.6002(0.5884, 0.6122) | <0.01 |
| 1977-1981 | 0.7818(0.7812, 0.7824) | <0.01 | 0.5033(0.4919, 0.5149) | <0.01 |
| 1982-1986 | 0.7145(0.7138, 0.7153) | <0.01 | 0.4121(0.4006, 0.4240) | <0.01 |
| 1987-1991 | 0.6318(0.6309, 0.6326) | <0.01 | 0.3145(0.3035, 0.3259) | <0.01 |
| 1992-1996 | 0.5445(0.5435, 0.5455) | <0.01 | 0.2266(0.2146, 0.2392) | <0.01 |
| 1997-2001 | 0.4574(0.4561, 0.4587) | <0.01 | 0.1640(0.1494, 0.1801) | <0.01 |
| 2002-2006 | 0.3874(0.3853, 0.3896) | <0.01 | 0.1254(0.1036, 0.1517) | <0.01 |
